# Prevalence and molecular determinants of colistin resistance among commensal Enterobacteriaceae isolated from poultry in northwest of Iran

**DOI:** 10.1186/s13099-019-0282-0

**Published:** 2019-01-31

**Authors:** Zeinab Pishnian, Mehri Haeili, Adel Feizi

**Affiliations:** 1 Department of Biology, Faculty of Natural Sciences, University of Tabriz, Tabriz, Iran; 2 Department of Clinical Sciences, Faculty of Veterinary Medicine, Islamic Azad University of Tabriz, Tabriz, Iran

**Keywords:** Colistin resistance, Food-producing animals, Gut microbiota

## Abstract

**Background:**

The emergence of colistin-resistant Enterobacteriaceae from human and animal sources is a public health concern as this antibiotic is considered to be the last line therapeutic option for infections caused by multidrug-resistant Gram-negative bacteria. Here we aimed to determine the prevalence of colistin resistance, among enterobacteria isolated from poultry and the possible underlying colistin resistance mechanisms.

**Methods:**

A collection of 944 cloacal samples were obtained from poultry and screened for colistin resistance. To uncover the molecular mechanism behind colistin resistance, the presence of plasmid encoded colistin resistance genes *mcr*-*1*, *mcr*-*2*, *mcr*-*3* and *mcr*-*4* was examined by PCR. The nucleotide sequences of the *mgrB*, *pmrA*, *pmrB*, *phoP*, *phoQ*, *crrA* and *crrB* genes were determined. The genetic relatedness of the colistin resistant (ColR) isolates was evaluated by Multilocus sequence typing. Three ColR mutants were generated in vitro by repetitive drug exposure.

**Results:**

Overall from 931 enteric bacteria isolated from poultry samples obtained from 131 farms, nine ColR bacteria (0.96%) with high level colistin resistance (MICs ≥ 64 mg/L) were detected all being identified as *K. pneumoniae.* The 9 ColR bacteria originated from different farms and belonged to 7 distinct Sequence types including ST11 (22.2%) and ST726 (22.2%) being the most prevalent STs followed by ST37, ST74, ST485, ST525 and novel sequence type 3380 (11.1% each). *mcr*-type genes were not detected in any isolate. In 88.8% of the isolates (n = 8), MgrB was inactivated by Insertion of IS elements (IS*1*-like, IS*3*-like, IS*5*-like families, positions + 75, + 113, + 117, + 135) and nonsense mutations at codons 8, 16, 30. All ColR isolates harboured wild type PmrA, PhoP, PhoQ or polymorphic variants of PmrB. Sequence analysis of the CrrB revealed a familiar S195N and 4 novel I27V, T150R, F303S and K325R substitutions. PmrB T93N substitution and *mgrB* locus deletion were identified in two laboratory induced ColR mutants and one mutant lacked alteration in the studied loci. In one ColR isolate with wild type MgrB an A83V substitution was detected in CrrA.

**Conclusion:**

It is concluded from our results that colistin resistance in the studied avian *K. pneumoniae* isolates was mostly linked to alterations identified within the *mgrB* gene.

## Background

Antibiotic resistance (ABR) rate has reached emergency levels both in human and veterinary medicine with many countries recognizing it as an important emerging threat to global public health and food safety. The agricultural use of human antibiotics as growth promoters or as prophylactic agents in farm animals has been criticized for contributing to the magnitude of the global challenge of ABR [1]. This is considered as a potential threat to human health as commensal resistant organisms propagated in food-producing animals can be transmitted to humans indirectly along the food chain or following direct contact or may serve as reservoirs of transferable antibiotic resistance determinants that could be transferred to human pathogens [1, 2]. For example, evidence supporting transmission of extended-spectrum beta-lactamase (ESBL) producing *Escherichia coli* clones, from livestock to human being presumably through the food chain [3]. Among the veterinary critically important antimicrobial agents is colistin -a last resort agent for treating multidrug-resistant (MDR) infections in humans- which is used mainly in poultry farms for prevention and treatment of Enterobacteriaceae infections [4]. Although not frequently used in human medicine, emergence of extensively drug-resistant (XDR) Gram-negatives bacteria (GNB), in clinical setting and paucity of novel marketed antibiotics, has led to the renewed interests in colistin for management of severe infections caused by these pathogens [5]. Polymyxins (polymyxin B and colistin) are bactericidal cationic antimicrobial peptides that exert their antibacterial effect mainly by disruption of outer membrane in GNB [6]. Resistance to this last-hope group of antimicrobials was recently detected among the members of Enterobacteriaceae in animals, retail meat and humans in china and subsequently reported by many regions of the world [7]. The most common mechanism of resistance among Enterobacteriaceae includes covalent modification of lipopolysaccharide (LPS) target through incorporation of positively charged groups. This alteration neutralizes the negative charges of the LPS and reduces binding affinity of cationic colistin to its target. LPS modifications in *Klebsiella pneumoniae* are known to be mediated mainly by genetic alterations in two component regulatory systems including PmrAB, CrrAB, PhoPQ or the negative feedback regulator of PhoPQ signaling system, MgrB [8–11]. Moreover, acquisition of plasmid encoded *mcr*-type genes, which encode enzymes able to modify LPS by the addition of phosphoethanolamine has been found to contribute in colistin resistance in most of the *E. coli*, *Salmonella* and some *K. pneumoniae* isolates [7, 12, 13].

Despite the fact that colistin has been widely used in veterinary medicine notably in poultry in Iran, little is known about the prevalence of colistin resistance and *mcr *genes among the commensal enteric bacterial isolates from food-producing animals in this geographic region. This study aimed to investigate the frequency of colistin resistance among commensal enteric bacteria isolated from poultry and to determine the possible underlying mechanisms among colistin-resistant isolates.

## Methods

### Screening for colistin resistant (ColR) enteric bacteria

A total of 944 laying hens and broilers as well as turkeys were studied between September 2017 to July 2018. Cloacal swabs were taken randomly from poultry referred to poultry diagnostic center (those with viral or metabolic diseases) of the northwest of Iran or from healthy broilers at one major chicken slaughterhouse (with process capacity of 25,000–40,000 chickens per day) located in Tabriz city, East Azerbaijan province. About 15 or three to five samples per farm were collected from the slaughterhouse or poultry disease clinic respectively and analyzed. The obtained samples (using sterile cotton swabs) were seeded on Eosin Methylene Blue (EMB) agar plates and were incubated at 37 °C for 24 h. All of the grown isolates were preliminary screened by inoculating the isolates into Mueller–Hinton broth (MHB) supplemented with colistin as follow: a suspension of the organism to be tested was prepared by suspending colonies from an overnight culture into the sterile MHB. The density of the suspension was measured with an Eppendorf Biophotometer and adjusted to OD600 of 0.085 (equivalent to the turbidity of a 0.5 McFarland standard). Three microliter of the prepared suspension were inoculated into two glass tubes containing 597 μL MHB supplemented with 2 and 4 mg/L colistin respectively to yield a final inoculum of ca. 5 × 10^5^ CFU/mL. The tubes were incubated in air at 37 °C for 16 to 20 h. An aliquot of 10 μL from all tubes in which visible bacterial growth was observed were seeded on EMB agar plates supplemented with 3 mg/L colistin which were then incubated for overnight at 37 °C. The isolated ColR bacteria were identified using conventional biochemical methods and were further tested for susceptibility to colistin by broth macrodilution method, whereby a minimum inhibitory concentration (MIC) of > 2 mg/L was considered indicative of colistin resistance according to the European Committee on Antimicrobial Susceptibility Testing (EUCAST) [14]. Colistin susceptible (ColS) *Pseudomonas aeruginosa* ATCC27853, *K. pneumoniae* ATCC 700603 and *E. coli* ATCC25922 were used as quality control for colistin susceptibility testing.

### Testing susceptibility to other antimicrobials

The susceptibility of the ColR isolates to various classes of antibiotics was determined by disk diffusion method (Kirby Bauer) according to the Clinical and Laboratory Standards Institute (CLSI (M100-S27) [15] guidelines using the following antibiotics: gentamicin, amikacin, ceftriaxone, ceftazidime, ceftazidime-clavulanate, imipenem, ciprofloxacin, levofloxacin, doxycycline, and fosfomycin (BBL Sensi-Disc™, Becton–Dickinson, Sparks, MD).

### Genotyping by multilocus sequence typing

Multilocus sequence typing (MLST) was carried out with seven standard housekeeping genes (*rpoB*, *gapA*, *mdh*, *pgi*, *phoE*, *infB *and *tonB*) according to method of Diancourt et al. [16]. Allele sequences and sequence types (STs) were analyzed by using the *K. pneumoniae* MLST database provided by the Institut Pasteur, Paris, Franc (http://bigsdb.pasteur.fr/klebsiella/klebsiella.html). To investigate the evolutionary relationship among our ColR isolates, BioNumerics (version 7.6.3; Applied Maths, Sint-Martens-Latem, Belgium) was used to construct a minimum spanning tree based on the MLST profiles.

### Identification of colistin resistance determinants

The chromosomal Deoxyribonucleic acid (DNA) of the avian isolates was extracted by the boiling method (https://www.eurl-ar.eu/CustomerData/Files/Folders/21-protocols/278_mcr-multiplex-pcr-protocol-v2-oct16.pdf) and were subjected for polymerase chain reaction (PCR) amplification of the genes encoding the PmrA, PmrB, MgrB, PhoP, PhoQ, CrrA and CrrB proteins using primers described previously [17] (primers pmrB-F1 and pmrB-R2 were used for PmrB). For amplification of *mgrB* locus two pairs of primers were used: (i) mgrB-F and mgrB-R (external to *mgrB*) targeting amplification of *mgrB* coding sequence as well as some flanking regions [17] (ii) Int_mgrB_F (5′-CGGTGGGTTTTACTGATAGTCA-3′) and Int_mgrB_R (5′-ATAGTGCAAATGCCGCTGA-3′) targeting amplification of an internal region of the *mgrB *coding sequence (used with some strains) [18]. The primers used for amplification of entire *crrA* and *crrB* genes were as follows: CrrA-F (5′-GCATGTTGTCATCAGCACTGTG-3′) and CrrA-R (5′-GGAACCGAGTATTGCAATGG-3′), CrrB-F (5′-GGATTGAAGGGCATTCCGGA-3′) and CrrB-R (5′-GCAGTATGTGGGATCTGTCT-3′). The PCR products were sequenced and the nucleotides and deduced protein sequences were analyzed at the National Center for Biotechnology Information web site. In addition, the *pmrA*, *pmrB*, *mgrB*, *phoP*, *phoQ*, *crrA* and *crrB* sequences of two ColS *K. pneumoniae* isolates obtained from healthy broilers in slaughterhouse were determined as wild-type controls. The IS Finder database (https://www-is.biotoul.fr/) was used to identify and analyze insertion sequences. Identified novel amino acid substitutions were further analyzed by using the Protein Variation Effect Analyzer tool (PROVEAN) (http://provean.jcvi.org/index.php) to predict their impact on the biological function of the protein (i.e. neutral or deleterious) [19]. All ColR isolates were also screened for the presence of plasmid encoded colistin resistance genes *mcr*-*1*, *mcr*-*2*, *mcr*-*3* and *mcr*-*4* genes by PCR as described previously [7, 13, 20, 21].

### Laboratory induction of colistin resistance

To assess whether colistin non-susceptibility occurs after colistin exposure, an in vitro resistance induction experiment was performed by exposing two ColS isolates obtained from healthy broilers to elevated concentrations of colistin. The EMB agar plates supplemented with 2 mg/L colistin were inoculated with 3 × 10^5^ CFU/mL CloS bacterial suspension and were incubated for 48–72 h. Colonies growing on each plate were picked and subcultured on media with the same or increasing colistin concentration (5, 7, 10, 20, 50 mg/L). Colistin MIC and susceptibility to other antibiotics in the selected mutants were determined by broth macrodilution and disk diffusion methods respectively.

## Results

### Bacterial screening, antimicrobial susceptibility testing and bacterial genotyping

**Fig. 1:**
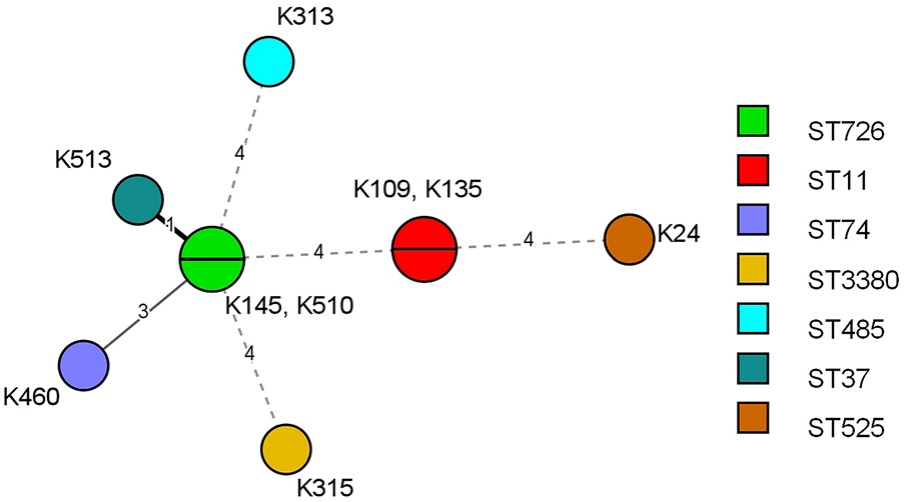
Minimal spanning tree (MST) of ColR *K. pneumoniae* isolated from poultry. Each circle corresponds to one ST and is subdivided into sectors corresponding to the number of isolates represented with this ST. The numbers on the connecting lines between STs and next to each circle correspond to the numbers of differing loci between the STs and strain’s ID characterized with specific ST respectively

Overall 944 samples were collected from 131 farms located in 29 different cities of the East Azerbaijan province which included 503 samples from healthy broilers at slaughterhouse and 441 samples from dead poultry referred to clinic during 2017–2018 (Table 1). A total of 931 isolated enteric bacteria (495 and 436 isolates from slaughterhouse and clinic respectively) were screened for colistin resistance according to cut off point of > 2 mg/L as defined by EUCAST. Overall, nine ColR enteric bacteria with MIC > 2 mg/L were detected all being identified as *K. pneumoniae* according to biochemical methods (excluding the number of *Proteus* spp. (n = 146) which are intrinsically resistant to polymyxins). All ColR isolates were characterized with high level colistin resistance with MICs ≥ 64 mg/L. The nine ColR *K. pneumoniae* (CRKP) isolates originated from different farms and belonged to 7 distinct Sequence types including ST11 (22.2%) and ST726 (22.2%) being the most prevalent STs followed by ST37, ST74, ST485, ST525 and novel sequence type 3380 (11.1% each) (Fig. 1). About 88.8% (n = 8) of ColR isolates showed resistance to at least one tested antimicrobial agent, including doxycycline (n = 8, 88.8%), levofloxacin (n = 7, 77.7%), ciprofloxacin (n = 7, 77.7%), gentamicin (n = 3, 33.3% resistant and n = 2, 22.2% with intermediate susceptibility) and fosfomycin (n = 1, 11.1%). No resistance was found for amikacin, ceftriaxone, ceftazidime and imipenem. The two ColS control *K. pneumoniae* isolates obtained from healthy broilers originated from different farms and cities and characterized with different colistin MIC values (0.25 and 1 mg/L). Doxycycline resistance was found in both ColS isolates among which one was characterized with, ciprofloxacin/levofloxacin and fosfomycin resistance as well. The colistin resistance induction assay was performed using these two ColS isolates to assess the contributing role of colistin exposure as a risk factor for colistin resistance emersion. The laboratory induced ColR isolate, K113R was obtained by six selection cycles in the presence of increasing concentrations of colistin. The colistin MIC of this isolate was found to be 32 mg/L which was 32 times higher than that in the progenitor ColS isolate K113S (MIC = 1 mg/L). Similarly, seven and six selection cycles were required to obtain two ColR mutants K79R1 (MIC > 128 mg/L) and K79R2 (MIC = 64 mg/L) respectively originated from a ColS isolate K79S (MIC = 0.25 mg/L). Indeed, from colonies originated form K79S grown on EMB agar supplemented with 2 mg/L colistin, two distinct colonies were picked and exposed separately to increasing colistin concentrations. Colistin resistance induction was not associated with change in susceptibility to other antimicrobials in laboratory induced ColR isolates (Table 2).

**Table 1:** Characteristics of the studied poultry samples

Sampling source	Number of samples (number of isolates)			City number	Farm number	Number of ColR isolates
	Laying hens	Broilers	Turkeys			
Poultry diagnostic center	30 (29)	388 (385)	23 (22)	21	98	4
Slaughterhouse	–	503 (495)	–	18	33	5
Total	30 (29)	891 (880)	23 (22)	29^a^	131	9

^a^Indicates the total number of unique cities by excluding the cities (n = 10) which were common between the two sampling sources

**Table 2:** Features of the studied colistin resistant and susceptible *K. pneumoniae* isolated from poultry

Isolate ID	Col MIC (mg/L)	Origin	Year of isolation	City	MgrB	PmrB	CrrA	CrrB	Sequence type	AMR
K135	> 128	Dead broiler	2017	Sardrud	WT	R256G	A83V	WT	ST11	D, CIP, LVX, GM^a^
K313	128	Dead broiler	2018	Marand	Insertion of IS*1*-like between + 116, + 117	R256G	WT	K325R	ST485	D, CIP, LVX
K315	> 128	Dead broiler	2018	Marand	Q30STOP	R256G	WT	S195N, I27V	ST3380	D
K513	> 128	Dead turkey	2018	Ajabshir	Insertion of IS*3*-like between + 112, + 113	R256G	WT	F303S, I27V	ST37	D, GM^a^, CIP, LVX
K24	> 128	Healthy broiler	2018	Malekan	L8STOP,∆c25	WT	WT	WT	ST525	D, CIP, LVX
K109	> 128	Healthy broiler	2018	Marand	Insertion of IS*5*-like between + 74, + 75	R256G	WT	WT	ST11	D, GM, CIP, LVX
K145	> 128	Healthy broiler	2018	Malekan	C16STOP	R256G	WT	T150R	ST726	D, GM, LVX, CIP, FOS
K460	64	Healthy broiler	2018	Bostanabad	Insertion of IS*3*-like between + 134, + 135	A246T	WT	WT	ST74	Non
K510	128	Healthy broiler	2018	Bostanabad	Q30STOP	R256G	WT	WT	ST726	D, GM, CIP, LVX
K79S	0.25	Healthy broiler	2018	Hashtroud	WT	R256G	WT	WT	ND	D, CIP, LVX, FOS
K79R1^c^	> 128	–	–	–	∆*mgrB* locus^b^	–	–	–	–	–
K79R2^c^	64	–	–	–	–	–	–	–	–	–
K113S	1	Healthy broiler	2018	Bostanabad	WT	WT	WT	K325R	ND	D
K113R^c^	32	–	–	–	–	T93N	–	–	–	–

*D* doxycycline, *CIP* ciprofloxacin, *LVX* levofloxacin, *GM* gentamicin, *FOS* fosfomycin, *WT* wild type, *ND* not determined, *AMR* antimicrobial resistance profile

(–) Characteristics similar to progenitor ColS isolate

^a^Intermediate susceptibility

^b^∆*mgrB* locus, not amplifiable with all primers used in this study

^c^Laboratory induced ColR isolate

### Molecular determinants of acquired colistin resistance

In order to analyze the molecular mechanisms accounting for colistin-resistance in the studied commensal enteric bacteria the presence of *mcr*-*1*, *2*, *3*, *4* genes were investigated in all isolates. As could be expected form the level of resistance (high), *mcr*-type genes were not detected in any isolate and therefore, presence of mutations in chromosomally encoded genes was suspected. Among the studied seven proteins, MgrB, a negative regulator of the PhoPQ signaling system, was the most altered protein being inactivated in 8 of 9 ColR avain isolates (88.8%). The MgrB inactivation was found to be mediated by two mechanisms: (1) insertion of IS elements belonging to 3 different families including IS*1*-like (768 pb bp), IS*5*-like [(ISEc68), 1197 bp] and IS*3*-like elements (1336 and 1259 bp) being inserted in different positions (+ 75, + 113, + 117, + 135) within the coding region of the protein. This inactivation mechanism was found in 4 isolates assigned to ST485, ST37, ST11 and ST74 genotypes and disrupted the coding region of *mgrB* and most likely mediated the resistance in these isolates (2) premature termination due to nonsense mutations at codon 30 [CAG (Gln) > TAG (Stop)], codon 8 [TTA (Leu) > TAA (Stop)] and codon 16 [TGC (Cys) > TGA (Stop)]. Moreover, a cytosine deletion at position + 25 was observed in MgrB harboring L8Stop substitution immediately after codon8, resulting in frame shifting and creation of the second and third stop codons TGA and TAG after codon 8. These nonsense mutations were observed in 4 isolates belonging to sequence types ST3380 (n = 1), ST525 (n = 1), ST726 (n = 2) and resulted in production of truncated proteins with 7, 15 and 29 amino acids instead of 47 amino acid wild-type MgrB. One ColR isolate with high level colistin resistance (MIC > 128 mg/L) assigned to ST11 carried a wild type MgrB and revealed a CrrA A83V (GCA > GTA) substitution as the only alteration in the studied genes. All ColR isolates revealed wild type *pmrA*, *phoP*, and *phoQ *genes. Sequence analysis of the CrrB revealed a common resistance conferring S195N (AGT > AAT) substitution and 4 novel amino-acid changes [I27V (ATA > GTA), T150R (ACG > AGG), F303S (TTT > TCT) and K325R (AAA > AGG)] in ColR *K. pneumoniae* isolates. PmrB R256G (CGC > GGC) and A246T (GCC > ACC) substitutions were also found in 8 ColR isolates. While all ColS isolates harbored wild type MgrB, PhoP, PhoQ, PmrA and CrrA, one isolate had a R256G mutated PmrB and the other had K325R substitution in the CrrB. In search of the altered gene in the laboratory induced ColR isolate K113R, during the repetitive exposure experiment, we found a novel T93N (ACC > AAC) substitution in the PmrB compared to ColS parent isolate. The isolate carried a wild type MgrB, PhoP, PhoQ, PmrA, CrrA and a K325R mutated CrrB similar to the progenitor ColS isolate (Table 2). More interestingly in laboratory induced ColR K79R1, which was characterized with > 512 times increase in colistin MIC after the induction assay, no amplification product was obtained using the external mgrB primers suggesting deletion of the *mgrB* locus. The absence of *mgrB* in the genome of this mutant was confirmed by PCR using the internal mgrB primers as described previously [18]. In ColR K79R2 isolate, no substitution was identified in the studied genes suggesting possible involvement of other unknown gene(s) in addition to the studied loci in conferring colistin resistance. While the PROVEAN prediction of the amino acid changes showed that PmrB T93N, CrrB F303S, T150R and CrrA A83V substitutions could have deleterious impact on the function of the corresponding proteins, CrrB K325R and I27V substitutions were categorized as neutral changes (Table 3).

**Table 3:** Predicting the impact of novel amino acid substitutions on the biological function of proteins using PROVEAN tool

Protein	Variant	PROVEAN score	Prediction (cutoff = − 2.5)	Isolates^a^
PmrB	T93N	− 3.38	Deleterious	ColR
CrrB	K325R	− 0.134	Neutral	ColR, ColS
CrrB	F303S	− 7.99	Deleterious	ColR
CrrB	I27V	− 0.055	Neutral	ColR
CrrB	T150R	− 5.99	Deleterious	ColR
CrrA	A83V	− 3.87	Deleterious	ColR

^a^Isolates in which the specific amino acid substitution has been identified

### Nucleotide sequence accession numbers

The nucleotide sequences of the mutated/altered *mgrB*, *crrB*, *pmrB *and *crrA *genes have been deposited at GenBank nucleotide sequence database under accession numbers MH990333 to MH990347.

## Discussion

The subtherapeutic application of antibiotics as prophylaxis or for growth promotion in farmed animals is described as a major contributor to the clinical problem of resistant disease in human medicine. There is evidence that food producing animals are likely contributor to the recent emergence of clinically important bacteria such as *mcr*-positive colistin resistant Enterobacteriaceae in human [22]. Colistin is among the most commonly used veterinary antibiotics in food animals worldwide and it is widely used in Iran for prevention/treatment of digestive tract diseases in chickens. In the current work we studied the prevalence and genetic background of ColR enteric bacteria colonizing the gut of poultry from 131 chicken farms located in 29 cities of East Azerbaijan province. We introduced here an effective screening method using selective broth media for accurate identification of ColR isolates from the polymicrobial samples such as cloacal swabs. It is important to note that alongside the screening using MHB supplemented with colistin, we performed the screening using the colistin containing agar plates (supplemented with 2 and 4 mg/L colistin) as well. However, since the agar dilution method has been found to produce higher colistin MIC values compared to broth dilution method [23, 24], we confronted with large number of false resistant cases. Moreover, there were some ColR isolate cases which were missed by agar screening method but detected by broth screening method (data not shown). From the 931 screened enteric bacterial isolates, 9 (0.96%) were found to be colistin resistant which were identified as *K. pneumoniae.* In this study we did not find any ColR *E. coli* isolate. ST11 was one of the most frequent Sequence types being found in 22.2% of ColR isolates originated from different farms and characterized with diverse resistance mechanisms. This sequence type has been previously reported by several other studies as a common ST among CRKP with human origin [11, 25, 26] indicating the successful circulation of this hypervirulent CRKP among the human and animal sources. One CRKP isolate obtained from turkey was characterized with sequence type 37 which has been frequently reported from CRKP of animal origin and is found to be associated with resistance to the last-hope anti-XDR-GNB antibiotics, colistin and tigecycline [27, 28]. In a search to unravel the colistin resistant mechanisms, we found that resistance in the studied CRKP isolates was not related to plasmid-encoded colistin resistance genes *mcr*-*1* to *mcr*-*4*. This was not a surprise for us as CRKP isolates studied here characterized with high MIC values (≥ 64 mg/L) which have been found to be caused by chromosomal alterations. On the other hand, *mcr*-type genes are suggested to confer a low level of colistin resistance. In 88.8% of the studied isolates, MgrB was inactivated by either insertion of IS elements or premature termination due to nonsense mutations. In four isolates belonging to sequence types ST3380, ST525 and ST726 a premature stop codon was identified in codons 8, 16 and 30 leading to production of truncated protein with 7, 15 and 29 amino acids respectively compared to wild-type MgrB (47aa). These amino acid substitutions were probably leading to production of a non-functional MgrB protein, and therefore were probably the source of colistin resistance. While Q30STOP substitution in CRKP had been previously reported by several other studies [10, 29], C16STOP and L8STOP were identified in our study as novel MgrB substitutions. In 4 isolates assigned to sequence types ST485, ST37, ST11 and ST74 *mgrB* was disrupted by insertion of mobile DNA belonging to three different IS*1*, IS*3* and IS*5*-like families. Surprisingly, all IS elements targeted 4 different sites within the *mgrB* gene. Several other studies have reported interruption of *mgrB* by IS*5*, and it was found that this mobile DNA always targeted the same location, between nucleotides 74 and 75 of the *mgrB *sequence [10, 18]. By comparing our results to other published data, it can be concluded that nucleotide positions + 74/+ 75 and + 88 (codon 30) in *mgrB *might constitute a hot spot of integration for ISs belonging to the IS*5* family or nonsense mutations respectively. Sequence analysis of PmrB revealed some variants including T246A and R256G in ColR isolates with the latter being also found in one ColS isolate. Since these alterations have been found in numerous ColS isolates [28, 29], they can’t contribute to colistin resistance alone and most likely correspond to polymorphisms as demonstrated previously [11, 17]. Overall, five amino acid substitutions were identified in CrrB, among which S195N had been previously described by Cheng et al. as contributing to colistin resistance [9]. Among the remaining 4 novel substitutions, only F303S and T150R were predicted by the PROVEAN tool to have deleterious impact on protein structure. We also suggest that amino acid change K325R in CrrB cannot contribute to colistin resistance alone since this mutation was also found in a ColS isolate. This substitution was also categorized as neutral change by the PROVEAN tool. One ColR isolate with high level colistin resistance (MIC > 128 mg/L) assigning to ST11 carried a wild type MgrB, CrrB, PmrAB, PhoPQ systems, but A83V substitution was detected in CrrA which might be harmful for protein as predicted by PROVEAN tool. In the three laboratory induced ColR mutants resistance was developed in a very short time period (6 or 7 selection cycles) indicating that colistin exposure might act as an important risk factor for resistance emersion. Sequence analysis of the studied genes revealed no alteration in the case of laboratory induced ColR mutant K79R2, implying that molecular factors other than what were studied here might mediate resistance in this isolate. PmrB T93N substitution and complete deletion of *mgrB* locus were identified in the other two laboratory induced ColR mutants. Cannatelli et al. previously reported CRKP isolates characterized with complete *mgrB* locus deletion which did not yield any PCR product when amplification was performed using different primers designed for flanking or internal sequences of *mgrB* [18]. Overall, whether novel changes recognized here influence colistin resistance is not known, as some have never been described before. The exact role of these novel variants in resistance conferring, require to be further studied by confirmatory tests, such as complementation assays with wild-type counterparts. However, according to previous studies demonstrating the key role of MgrB alterations in conferring colistin resistance, and observations regarding inactivation of *mgrB* gene in 88.8% of the avain CRKP isolates and induction of high level colistin resistance following *mgrB* locus deletion it can be concluded that colistin resistance in the studied avian CRKP isolates was mostly linked to alterations identified within the *mgrB* gene. We had previously reported MgrB inactivation as the major mechanism mediating colistin resistance in Iranian KP isolates of clinical origin indicating the fact that colistin resistance in CRKP isolates of human or animal sources is mediated by a common mechanism [17].

## Conclusion

We described here occurrence of colistin resistant *K. pneumoniae* with high level colistin resistance among the intestinal microbiota in poultry. We found MgrB alterations as the major mediator of colistin resistance in the studied avian CRKP isolates. Although we observed a low prevalence of ColR commensal bacteria (0.96%), there is always a potential for these isolates to easily spread across the country via the food chain or direct contact. The more worrisome scenario for these isolates would be acquiring resistance to carbapenems. Developing national programs to monitor antibiotic consumption and ABR in food animals and humans, are crucial for managing the concern of ABR spread via the food chain in the country.

## Abbreviations

ABR: antibiotic resistance
GNB: Gram negative bacteria
ColR: colistin resistant
ColS: colistin susceptible
MDR: multidrug-resistant
XDR: extensively drug-resistant
LPS: lipopolysaccharide
CRKP: colistin resistant *K. pneumoniae*
MLST: multilocus sequence typing
ST: sequence type
